# Delta-Globin Gene Expression Is Enhanced *in vivo* by Interferon Type I

**DOI:** 10.3389/fmed.2020.00163

**Published:** 2020-05-22

**Authors:** Maria Francesca Manchinu, Michela Simbula, Cristian Antonio Caria, Ester Musu, Lucia Perseu, Susanna Porcu, Maristella Steri, Daniela Poddie, Jessica Frau, Eleonora Cocco, Laura Manunza, Susanna Barella, Maria Serafina Ristaldi

**Affiliations:** ^1^Istituto Di Ricerca Genetica e Biomedica Del Consiglio Nazionale Delle Ricerche, Monserrato, Italy; ^2^Department of Medical Science and Public Health, Centro Sclerosi Multipla, University of Cagliari, Cagliari, Italy; ^3^Ospedale Microcitemico “A. Cao” - A.O. “G. Brotzu”, Cagliari, Italy

**Keywords:** erythropoiesis, δ*-globin* gene, interferon type 1, beta thalassemia, sickle cell anemia

## Abstract

Beta hemoglobinopathies are widely spread monogenic lethal diseases. *Delta-globin* gene activation has been proposed as a possible approach for curing these pathologies. The therapeutic potential of delta-globin, the non-alpha component of Hemoglobin A_2_ (α2δ2; HbA2), has been demonstrated in a mouse model of beta thalassemia, while its anti-sickling effect, comparable to that of gamma globin, was established some time ago. Here we show that the *delta-globin* mRNA level is considerably increased in a *Deoxyribonuclease II-alpha* knockout mouse model in which type 1 interferon (interferon beta, IFNb) is activated. IFNb activation in the fetal liver improves the *delta-globin* mRNA level, while the *beta-globin* mRNA level is significantly reduced. In addition, we show that HbA2 is significantly increased in patients with multiple sclerosis under type 1 interferon treatment. Our results represent a proof of principle that delta-globin expression can be enhanced through the use of molecules. This observation is potentially interesting in view of a pharmacological approach able to increase the HbA2 level.

## Introduction

Modulations of Fetal-hemoglobin (HbF) and possibly hemoglobin-A2 (HbA2) are of interest given their potential roles in ameliorating beta thalassemia (beta thal) and sickle cell anemia phenotypes (1–4).

Recently, through genome-wide association studies (GWASs) in the SardiNIA cohort, Danjou et al. identified new variants associated with levels of HbF, HbA1 (Hemoglobin A1), and HbA2 (5). In the regional association plots, at the loci associated with HbF reported by the authors (5), we noticed some suggestive, although not genome wide significant, signals covering a region on chromosome 19 were two genes related to erythropoiesis are present: *Krüppel-like factor 1 (Klf1)* and *Deoxyribonuclease II-alpha (DNase2a)*. The effect of Klf1 on HbF, HbA1, and HbA2 expression has been largely elucidated (6–9), while a possible effect of DNase2a on hemoglobins expression has not yet been investigated (10, 11).

DNase2a is expressed in the central macrophage of erythroblastic islands (CMEI), where it is involved in the digestion of extruded nuclei of developing erythrocytes (10, 12). *DNase2a* knockout (KO) mice die at around embryonic day 17 (E17) of lethal anemia, which is caused by IFNb production by macrophages (12). Undigested DNA directly stimulates CMEIs to express IFNb and, therefore, Interferon-responsive genes (12). *Ifnar1* KO rescues the impaired erythropoiesis of the *DNase2a* KO phenotype (12).

To investigate a possible effect of DNase2a on the expression of beta-like (*gamma, delta*, and *beta*) globin genes, we inter-crossed *DNase2a* KO mice with a transgenic mouse line (ln72) containing the full human *beta-globin* gene cluster (13). Expression of *globin* genes and erythropoiesis have been analyzed in fetal liver. Here we show that type I interferon activation led to a significant increase in the *delta-globin* mRNA level offset by a decrease in the *beta-globin* mRNA level and to a different pattern of erythroid differentiation compared to the control mice. No significant increase in the *gamma-globin* mRNA level was observed.

With the aim of verifying whether the use of type I interferon was able to modify the expression of HbA2 in humans, we conducted a study in patients with multiple sclerosis (MS) who underwent therapy with IFNb. Our results show a significant increase in HbA2 level in patients.

Beta hemoglobinopathies affect the health of countless people worldwide (14). At present, bone marrow transplantation provides the only definitive cure for these diseases. Alternative therapies such as gene therapy (15, 16) will be difficult to apply on a large-scale basis and in developing countries. Therefore, the development of a pharmacological approach for these pathologies would make care accessible in countries where these diseases are more widespread and mortality is very high (17–19).

Recently, we have validated the therapeutic potential of the *delta-globin* gene in a mouse model of beta thal (4). It is also well-known that HbA2 can inhibit Sickle hemoglobin (HbS) polymerization as efficiently as HbF (3).

Our results show, just as proof of principle, that HbA2 can be increased pharmacologically, and this observation could be a starting point for future studies aimed at increasing HbA2 levels through the use of molecules.

## Materials and Methods

### Mice

All experimental protocols were approved by the Cagliari University Institutional Animal Care and Use Ethical Committee (OPBA, Approval number: 22/2016). All methods were performed in accordance with relevant guidelines/regulations.

The original ln72 (provided by Dr. Frank Grosveld's laboratory) and the *DNase2a/Ifnar1* KO (bought from RIKEN BioResource Center, Japan) mouse lines were maintained on a hybrid C57BL/6 background.

### Genotyping

Genotypes were determined from genomic DNA by PCR.

Transgenic mouse line ln 72, an established single copy transgene that contains the full human beta-globin cluster (12), was genotyped using the primers listed in Supplemental Table 1.

WT and *Ifnar1* KO were detected with a wild-type-specific primer or mutant-specific reverse primer and a common forward primer.

WT and *DNase2a* KO were detected with a wild-type-specific or mutant-specific primer and an antisense primer. All primers are listed in Supplemental Table 1.

### Real-Time Quantitative PCR (RT-qPCR)

Total RNA was extracted from E12.5, E14.5, and E 16.5 fetal livers, or human tissue culture cells, using the RNeasy Mini Kit (Qiagen) as described by the manufacturer's protocol. The cDNA was made from total RNA using Superscript III reverse transcriptase (Invitrogen). RT-qPCRs were performed using SYBR Green chemistry (Applied Biosystems) with an ABI PRISM 7900 thermocycler (Applied Biosystems, Foster City, CA).

RT-qPCR was performed to measure the *gamma, beta*, and *delta globin* gene mRNA expression, and samples were normalized with respect to alpha mouse levels or HPRT human levels.

All primers are listed in Supplemental Table 1.

The reactions were performed on at least three different samples in triplicate for mice fetal liver and three times for two separate samples of human tissue culture cells. The analysis of RT-qPCR data was done using the ΔΔCT method.

### Flow Cytometry Analysis

Fetal liver cells were collected, from a minimum of three embryos per genotype, at 14.5 and 16.5 days post coitum (dpc). Cell suspensions were obtained, and isolated cells (1 x 10^6^ per sample) were stained with anti-mouse Ter119 FITC and anti-mouse CD71 PE antibodies (BD-Bioscience) at a final concentration 1:100. Cells were incubated for 20 min at 4°C, washed with PBS (5% BSA), and re-suspended in FACS buffer. A FACSCANTO (BD-Bioscience) flow cytometer was used to collect data and analyzed with FACSDiva software Version 6.1.3 (BD Biosciences) and FlowJo V7.6.5.

### Primary Human Erythroid Cultures

Human erythroid progenitor cells from peripheral blood were obtained from healthy individuals.

Donors cells were cultured using the two-phase liquid culture described by Fibach et al. (20) and Pope et al. (21) in the presence of 0, 10, or 100 UI IFNb 1a.

Written, informed consent was provided by the study participants.

### Patient Selection and Blood Sample Analysis

All experimental protocols were approved by the Ethics Committee ATS Sardegna (approval number 85/2018/CE). All methods were carried out in accordance with relevant guidelines and regulations. Written informed consent was obtained from all subjects.

A total of 81 Multiple Sclerosis patients were enrolled in the study from the Multiple Sclerosis Center (Binaghi Hospital, ATS Sardegna, Department of Medical Sciences and Public Health, University of Cagliari). Blood samples were collected from all patients in tubes containing EDTA anticoagulant for hemoglobin electrophoresis. Hemochrome was carried out by standard techniques. HbA2 levels were measured with high-performance chromatography.

Globin chain analyses were performed on a VARIANT II high-performance liquid chromatography system (Bio-Rad, Segrate MI, Italy). Two-level calibration of the instrument and sample analysis were carried out according to the manufacturer's recommendations. Types of interferon administrated during the study are listed in Supplemental Table 2.

### Statistics

In order to avoid problems related to non-normal distribution of values when applying statistical parametric tests (i.e., the *t*-test) to blood sample measurements, the inter-group difference was assessed with the non-parametric Wilcoxon signed-rank test (one-sided). In particular, differences in HbA2 levels between groups were assessed using the unpaired two-samples Wilcoxon test while, when comparing HbA2 levels before and after treatment, the paired samples Wilcoxon test was applied (Supplemental Table 3). Statistical power was calculated with a Wilcoxon-Mann-Whitney test for two groups at a significance of 0.05, one-sided.

Otherwise, statistical differences were calculated using the unpaired Student's *t*-test.

*P*-value < 0.05 was considered statistically significant, and a Bonferroni correction for multiple testing was applied when appropriate.

The statistical analyses were performed using R (http://www.Rproject.org) and G^*^Power Version 3.1.9.2.

## Results

### Human *Delta-Globin* Gene Expression Is Increased in *DNase2a*-Deficient Mouse Fetal Liver

In this study, we aimed to evaluate the possible effect of *DNase2a* deficiency on human *beta-like globin* gene expression *in vivo*. To this end, we crossed a transgenic line containing the entire human *beta-globin* gene locus (ln72) (13) with the *DNase2a* KO mouse model (12).

Since mice deficient in *DNase2a* die around E17, we evaluated *beta-like globin* mRNA levels in fetal liver at 12.5, 14.5, and 16.5 dpc.

**Figure 1:**
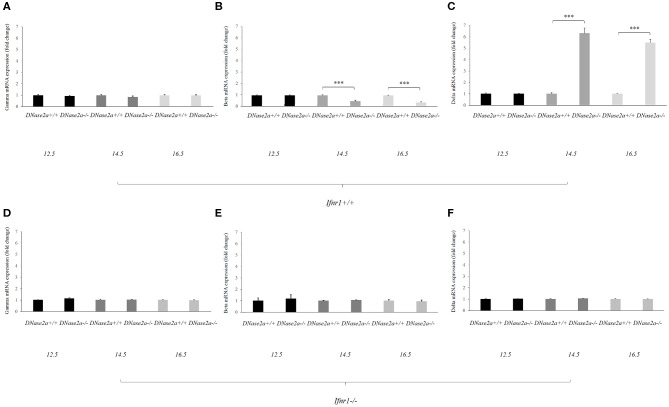
Human delta-globin gene expression is increased in *DNase2a*-deficient mouse fetal liver. **(A–C)** represent the mRNA expression level of the human *gamma, beta*, and *delta globin* genes, respectively, in *DNase2a* ^+/+^ *Ifnar1*^+/+^ (*n* = 4 for each time point) and *DNase2a* ^−/−^ *Ifnar1*^+/+^ (*n* = 4 for each time point) fetal liver at 12.5, 14.5, and 16.5 dpc. **(D–F)** represent the expression levels of the human *gamma, beta*, and *delta globin* genes, respectively, in *DNase2a* ^+/+^ *Ifnar1*^−/−^ (*n* = 4 for each time point) and *DNase2a*^−/−^ *Ifnar1*^−/−^ (*n* = 4 for each time point) fetal liver at 12.5, 14.5, and 16.5 dpc. Levels of significance, calculated by Student's *t*-test, are indicated. ***p < 0.001.

No effect on the *gamma-globin* mRNA level was detected (Figure 1A).

The *beta-* and *delta-globin* mRNA levels at 12.5 dpc were comparable in WT and *DNase2a*-deficient mouse fetal livers (Figures 1B,C).

At 14.5 dpc, the *beta-globin* mRNA level was diminished in *DNase2a* KO with respect to WT fetal liver (0.48 ± 0.07, *p* = 8.93 x 10^−5^). The decreased level of *beta-globin* mRNA was also observed at 16.5 dpc (0.37 ± 0.06, *p* = 5.57 x 10^−5^) (Figure 1B).

At the same time the *delta-globin* mRNA level was increased in *DNase2a* KO embryos with respect to WT embryos at 14.5 dpc (6.15 ± 0.4, *p* = 2.30 x 10^−4^) and 16.5 dpc (5.34 ± 0.2, *p* = 1.09 x 10^−5^) (Figure 1C).

As a control, we crossed ln72 mice to *DNase2a/Ifnar1* double KO mice. Embryos with a double deficiency for *DNase2a* and *Ifnar1* do not show differences in the expression of the human globin genes with respect to WT (Figures 1D–F).

These results indicated that IFNb affects the levels of *beta-* and *delta-globin* mRNAs. Starting from day 14, when definitive erythropoiesis definitely takes place, the *delta-globin* mRNA level is increased (6.15- and 5.34-folds at 14.5 and 16.5 dpc, respectively) while the *beta-globin* mRNA level is significantly reduced (0.48- and 0.37-fold at 14.5 and 16.5 dpc, respectively).

The mouse alpha-globin mRNA level is not affected by DNase2a deprivation (Supplemental Figure 1).

### Fetal Liver Erythropoiesis in *DNase2a*-Deficient Mouse

**Figure 2:**
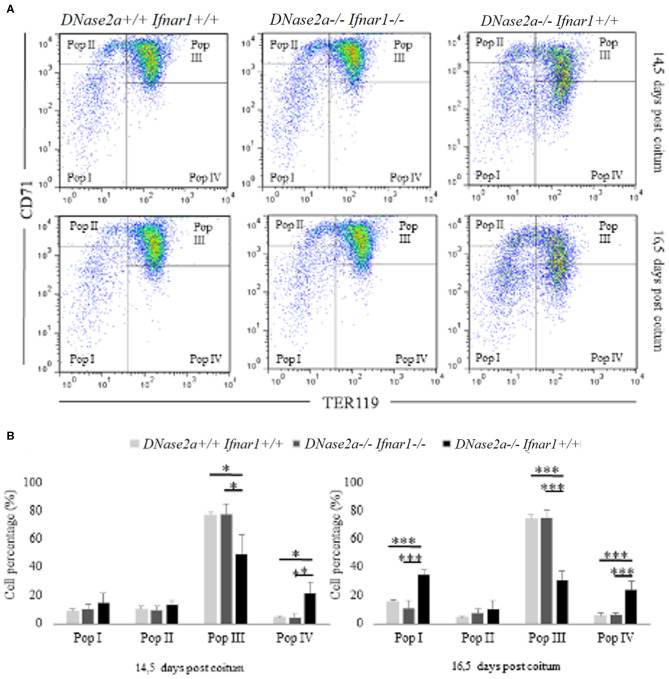
Flow cytometry analysis of fetal liver cells from 14.5 and 16.5 dpc embryos. In panel **(A)**, Pop. I, Pop. II, Pop III, and Pop IV were gated (according to levels of expression CD71 and TER119) and analyzed for each genotype studied at 14.5 dpc (upper panel) and at 16.5 dpc (bottom panel) in fetal liver cells. **(B)**: Bar plots showing flow cytometry analysis from 14.5 dpc (*n* = 4; left panel) and 16.5 dpc (*n* = 4; right panel) fetal liver cells. Levels of significance, calculated by Student's *t*-test, are indicated. *p < 0.05; **p < 0.01; ***p < 0.001.

In *DNase2a*-deficient mice, definitive erythropoiesis is impaired due to IFNb activation (12). However, fetal liver erythropoiesis in *DNase2a* KO mice has never been analyzed by flow cytometry before. To evaluate whether the observed increase in *delta-globin* gene expression could be somewhat correlated to a modification of the normal erythropoietic kinetics (22), we analyzed, through flow cytometry, fetal liver definitive erythropoiesis. Analysis was conducted on WT, *DNase2a* KO, and *DNase2a/Ifnar1* double KO freshly isolated fetal liver cells from 14.5 and 16.5 dpc mice embryos according to levels of expression of TER119 and CD71 (23) (Figure 2A). We excluded from the analysis all events that expressed neither TER119 nor CD71, since only cells in the erythroid lineage were considered. Four different states of maturation were analyzed: Pop. I (TER119 low or absent/CD71 low or absent), Pop. II (TER119 low or absent/CD71 high), Pop. III (TER119 high/CD71high), and Pop. IV (TER119 high/CD71 low or absent). No significant differences were observed in the frequency, morphology, and levels of expression of Ter119 and CD71 in maturing erythroid cells between WT and *DNase2a/Ifnar1* double KO mice fetal livers at 14.5 dpc or at 16.5 dpc (Figure 2B). On the other hand, analysis of mice lacking *DNase2a* gene displayed different frequencies of the maturing cells (Figure 2B). Analysis showed a significant increase in Pop I in 16.5 dpc (WT: 15.6% ± 1.44; *DNase2a KO*: 34.5% ± 3.4 in 16.5 dpc, *P* = 1.79 x 10^−5^). No significant difference in the percentage of Pop. II was detected, while a significant reduction of Pop. III was observed (WT: 76.82% ± 2.37; *DNase2a KO*: 49.53% ± 13.31 in 14.5 dpc, *P* = 0.0032, WT: 74.45% ± 2.86; *DNase2a KO*: 30.98% ± 6.22 in 16.5 dpc, *P* = 4.13 x 10^−6^). A significant contemporary increase in Pop. IV was registered in the *DNase2a* KO genotype in comparison to WT (WT: 4.21% ± 1.09; *DNase2a* KO: 21.5% ± 7.63 in 14.5 dpc, *P* = 0.001, and WT: 5.64% ± 2.22; *DNase2a* KO: 23.98% ± 6.03 in 16.5 dpc, *P* = 7.3 x 10^−4^).

Taken together, our data show a variation in the erythropoietic pattern of differentiation in mice lacking *DNase2a* gene, with a decreased frequency of Pop. III and an increased frequency of later populations (Pop. IV).

An increased frequency of Pop I, containing the earliest erythroid progenitors (21), is observed in the 16.5 dpc *DNase2a* KO. This increase, which is not observed in the 14.5 dpc KO mice, is most likely explained by the anemia that becomes progressively more and more severe starting from the establishment of definitive erythropoiesis in the fetal liver of *DNase2a* KO mice (12). The observed varied pattern of erythroid differentiation in *DNase2* KO fetal liver is, however, distinct from that observed in the ineffective erythropoiesis seen, for example, in beta thal, which is characterized by a decrease in the number of the later population (Pop IV) and an increased number of earlier populations (Pop I, II, and III) (24, 25).

### Hemoglobin A2 Levels Increase After IFNb Treatments in Multiple Sclerosis Patients

In this study, we have shown that the *delta-globin* mRNA level is increased in *DNase2a* null fetal liver at 14.5 and 16.5 dpc. We have also shown that higher *delta-globin* mRNA is the consequence of type 1 interferon (IFN1) activation.

To evaluate the effect IFNb on the *delta-globin* mRNA level in human erythroid cells, we carried out human erythroid progenitor liquid culture (20, 21) from two healthy donors. The relative normalized mRNA level of the human *delta* vs. *beta-globin* genes at 12 and 14 days after stimulation with 0, 10, or 100 UI of IFNb 1a was evaluated. The results did not show statistical differences in the expression of the *delta*-*globin* gene in either of the two independent cultures analyzed (Supplemental Figure 2).

It has been reported that multiple sclerosis patients treated with IFNb have higher levels of HbA2 in comparison to those treated with other drugs (26). For the purpose of verifying whether therapy with IFNb was the cause of the increase in HbA2 *in vivo*, we performed a study in MS patients.

First, a transversal study was carried out on 47 MS patients undergoing IFNb therapy for at least 1 year; HbA2 average did not show any difference compared to the control population (2.70% ± 0.26 vs. 2.71% ± 0.32). Since a compensatory mechanism could occur in the erythropoiesis of patients treated for a long period, we carried out a longitudinal study (*n* = 25) analyzing HbA2 levels at the diagnosis of the disease (T0) and after 3 months of drug treatment (T1). HbA2 average in MS patients before IFNb treatment (T0) was 2.75% ± 0.71, while after treatment, it was 2.87% ± 0.80. Blood counts were within normal limits for all patients, and no significant differences were detected between at T0 and T1 (Supplemental Table 4).

Interestingly, in a beta thal carrier patient, the effect of interferon therapy was found to be the highest in all of the studied samples, from 5.9% (T0) to 6.4% (T1) g/dl.

**Figure 3:**
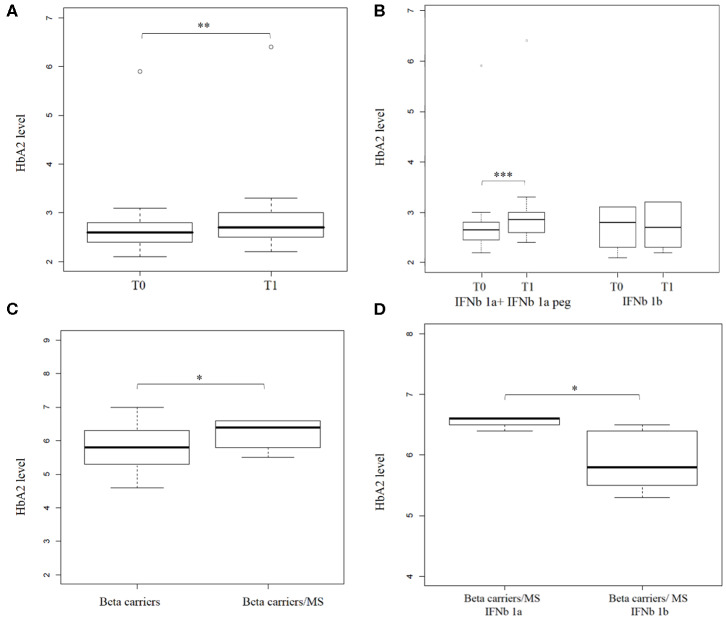
Hemoglobin A2 levels increase after interferon beta (IFNb) treatment in Multiple Sclerosis (MS) patients. **(A)** shows a box plot of HbA2 levels in (*n* = 25) MS patients before (T0) and after (T1) IFNb treatment. **(B)** shows a box plot of HbA2 level in (*n* = 22) MS patients before (T0) and after (T1) IFNb treatment stratified by IFNb type (16 and 6 patients under IFNb 1a + IFNb 1a peg and IFNb 1b treatment, respectively). **(C)** shows a box plot of HbA2 levels in beta thal carriers (*n* = 643) and in beta thal carriers/MS patients under IFNb treatment (*n* = 9). **(D)** shows a box plot of HbA2 levels determined in beta thal carriers/MS patients stratified by IFNb type (4 and 5 patients under IFNb 1a and IFNb 1b treatment, respectively). Levels of significance, calculated by Wilcoxon test, are indicated. *p < 0.05; **p < 0.01; ***p < 0.001.

The box plot in Figure 3A shows HbA2 before and after treatment, and the Wilcoxon test revealed a significant difference in HbA2 levels (*p* = 1.6 x 10^−3^, one-sided). Stratification of the sample according to the type of IFNb administrated (IFNb 1a or IFNb 1b, see Supplemental Table 2) revealed that this difference is mainly due to IFNb 1a (IFNb 1a + IFNb 1a peg) treatment. HbA2 in patients before treatment with IFNb 1a was 2.8% ± 0.8, while after treatment, it was 3.02% ± 0.9 (*p* = 1.8 x 10^−3^, one-sided), as shown in Figure 3B.

A significant number (*n* = 9) of beta thal carriers were represented in our transversal study, so we analyzed them separately. Average HbA2 in beta thal carriers/MS patients was 6.24% ± 0.42 compared to 5.84% ± 0.53 in beta thal carriers.

The box plot in Figure 3C shows the HbA2 levels in the two different carrier groups; the Wilcoxon test revealed a significant difference in HbA2 levels (*p* = 0.01 one-sided). Stratification of the sample revealed, as before, that this difference is mainly due to IFNb 1a treatment. The HbA2 value in carriers of beta thal treated with IFNb 1a was 6.55% ± 0.1, while that of those treated with IFNb 1b was 5.9%± 0.5 (*p* = 0.023, one-sided), as shown in Figure 3D.

Our results suggest that IFNb induced a significant increase in Hemoglobin A2 levels in patients with MS. Moreover, this effect seems to be more relevant and persistent in beta thal carriers/MS.

## Discussion

In this study, we have investigated the effect of DNase2a deprivation on globin gene expression and erythropoiesis *in vivo*. In *DNase2a* null fetal liver, the *delta-globin* mRNA level is increased while the *beta-globin* mRNA level is significantly reduced. Erythropoiesis is altered with an increased frequency of the Ter 119 high/CD71 low or absent cell population (Population IV) (23).

In definitive terminal erythropoiesis, in humans, the peak of *delta-globin* expression appears in earlier stages compared to that of *beta-globin*, and it tends to drop in enucleated circulating reticulocytes and RBCs (3). A similar effect occurs with regards to earlier *delta-globin* expression in the beta-locus transgenic mice used in the present study (Supplemental Figure 3). Since reticulocytes have little *delta-globin* mRNA, it can be deduced that the observed increase is likely due to the increased proportion of Pop. IV, which is mainly composed of orthochromatic erythroblasts (23).

The asynchronous synthesis of beta and delta chains during erythroid maturation is most probably due to a mechanism linked to the activation of the promoter based on the proximity to the LCR for which the closest promoter is activated earlier, or more frequently, and then stabilized at the *beta-globin* gene promoter (22, 27). Another contributing factor is the different stability of the *beta-* and *delta-globin* mRNAs (28). The combination of these mechanisms would explain the higher level of *delta* mRNA in earlier populations.

The observed altered pattern of erythroid differentiation and increase in *delta-globin* mRNA level is due to type I interferon activation in the fetal liver, as demonstrated by the fact that erythropoiesis and globin gene expression are rescued to those of WT in *DNase2a/Ifnar1* double KO mice. In support of this notion, there is also the observation that there is a small but significant increase of HbA2 in patients affected by MS and undergoing IFNb therapy. The increase appears to be more consistent and durable in beta thal carrier MS patients. The effect is primarily due to the increase in HbA2 levels in patients subjected to therapy with interferon-beta1a (IFNb 1a, produced by mammalian cells), whereas interferon-beta1b (IFNb 1b, produced by genetically modified E. coli) does not seem to have an effect, at least with the doses used for MS. This difference may be due to the difference in biological activity between the two drugs, with IFNb 1a being ten times more active than IFNb 1b (29), as well as to the difference in dose and frequency of administration of the two types of interferon (30). Despite the low number of MS patients affected by beta thal (9 subjects) with respect to non-MS beta thal patients [data derived from Danjou et al. (5)] available for this study, the difference we observed was supported by a moderate statistical power (52%). Even stronger was the power to observe a significant difference in HbA2 levels between beta thal MS patients treated with INFb 1a and 1b (67%), further supporting the robustness of our results. However, it would be interesting to assess the effect on HbA2 levels of IFNb 1a in carriers of beta thal not affected by MS, though this would require a dedicated clinical study.

In humans, only three patients homozygous for a *DNase2a* null mutation have been described in the literature (31). In these patients, type one interferon is activated. An effect on erythropoiesis with mild anemia, especially at birth, has been described (28). We had the chance to test mRNA levels for *delta-globin* in one of these patients before and after treatment, which strongly ameliorated patient condition (31). A significantly different level of *delta-globin* mRNA was detected, with a 30% (±4.15%) higher *delta-globin* mRNA level before treatment than after treatment (*p* = 0.031, technical triplicate). These data, although limited to a single patient, suggest that in humans, the activation of type 1 interferon affects erythropoiesis (31), with effects on the *delta-globin* mRNA level.

The increase in *delta-globin* represents an alternative experimental approach for the treatment of beta thal and sickle cell anemia (3, 4). A therapeutic strategy for beta thal and sickle cell anemia based on the increase in *delta-globin* may have the advantage over reactivating *gamma-globin* that the expression of the *delta-globin* gene is pan-cellular, while that of the *gamma-globin* gene is heterocellular (3). Moreover, the oxygen affinity of HbA2 is more similar to that of HbA1 than is HbF. In this work, we also observed that, as a consequence of interferon activation, the increased *delta-globin* mRNA level corresponds to a decrease in the *beta-globin* mRNA level. In sickle cell anemia, this may represent a further advantage.

Several drugs are under investigation as HbF-inducing agents. However, up to today, only Hydroxyurea is utilized in the clinic, with many limitations (32). Other strategies for the reactivation of the *gamma-globin* gene have been based on interventions aimed at modifying hemoglobin switching through genomic modification (1, 2). Similar approaches have been proposed for the *delta-globin* gene (4, 33). These strategies, however, are in their infancy, and several issues concerning safety and efficacy have to be addressed before translating these approaches to the clinic.

It is difficult to predict what increase in HbA2 could be achievable with a targeted pharmacological approach. In the case of sickle cell anemia, HbA2 above 10% (about 4 times the normal level) of total hemoglobin would be beneficial. Levels above 30% (about 12 times the normal level) should be curative (34, 35). These predictions are based on the anti-sickling properties of HbA2, which are similar to those of HbF (3). On the other hand, higher increases would be needed for an improvement in beta thal major. However, even if the achievable increase was not enough to cure the diseases in combination with other globin therapies, this may be a contributing factor to improve patient condition.

The precise molecular pathway by which IFNb causes a perturbation of terminal erythroid maturation, with an increase in Pop IV, and of *delta-globin* mRNA is not fully elucidated and needs further investigation. Most likely, however, the observed change in erythroid maturation kinetics is in part due to the perturbation of the apoptotic program, necessary to terminal erythroid differentiation (36, 37), caused by IFNb (38).

We are aware that repositioning of IFNb for beta hemoglobinopathies is unlikely, since type 1 interferon has been universally known as a lethal inhibitor of erythropoiesis (39, 40). Other pathways that may affect the erythropoietic cell cycle kinetics should be investigated. In this regard, it is interesting to note that CCND3, a D-type cyclin that coordinates the cell cycle during erythroid differentiation (41), was found to be associated with increased HbA2 levels in a recent study (5). *CCND3* gene product cyclin D3 plays a critical role in regulating the number of cell divisions that erythroid precursors undergo during terminal differentiation (41). CCND3 null mice are viable and fertile and do not show important signs of anemia (41). These observations suggest that there could be a viable pathway to alter the cell cycle during terminal erythroid differentiation, as happens in CCND3 KO mice, through the use of molecules and without serious pathological consequences. The molecular mechanism through which CCND3 affects *delta-globin* gene expression remains, however, to be more clearly determined.

In summary, our study represents “a proof of principle” that elevation of delta-globin could be an interesting target for a pharmacological approach aimed at the therapy of beta hemoglobinopathies.

## Data Availability Statement

All datasets generated for this study are included in the article/Supplementary Material.

## Ethics Statement

The studies involving human participants were reviewed and approved by Ethics Committee ATS Sardegna (approval number 85/2018/CE). The patients/participants provided their written informed consent to participate in this study. The animal study was reviewed and approved by Cagliari University Institutional Animal Care and Use Ethical Committee (OPBA, Approval number: 22/2016).

## Author Contributions

MM, MSi, CC, EM, LP, MSt, and DP performed the experiments and contributed to the interpretation of the results. EC and JF supervised the collection of samples and clinical data from patients. SB performed hematological evaluation of patients. LM carried out human erythroid progenitor liquid culture. MM, MSi, CC, and SP contributed to the writing of the manuscript and prepared the figures. MR and MM designed the study. MR designed the study, supervised the research, and wrote the manuscript. All authors contributed to the discussion and approved the final manuscript.

## Conflict of Interest

The authors declare that the research was conducted in the absence of any commercial or financial relationships that could be construed as a potential conflict of interest.
