# Supplementary material for: Delta-Globin Gene Expression Is Enhanced in vivo by Interferon Type I
Source: Front Med (Lausanne). 2020 May 22;7:163. doi: 10.3389/fmed.2020.00163 (PMC7256663; doi:10.3389/fmed.2020.00163)
Supplement: Supplementary file 1 [file Data_Sheet_1.PDF]

Supplementary Material

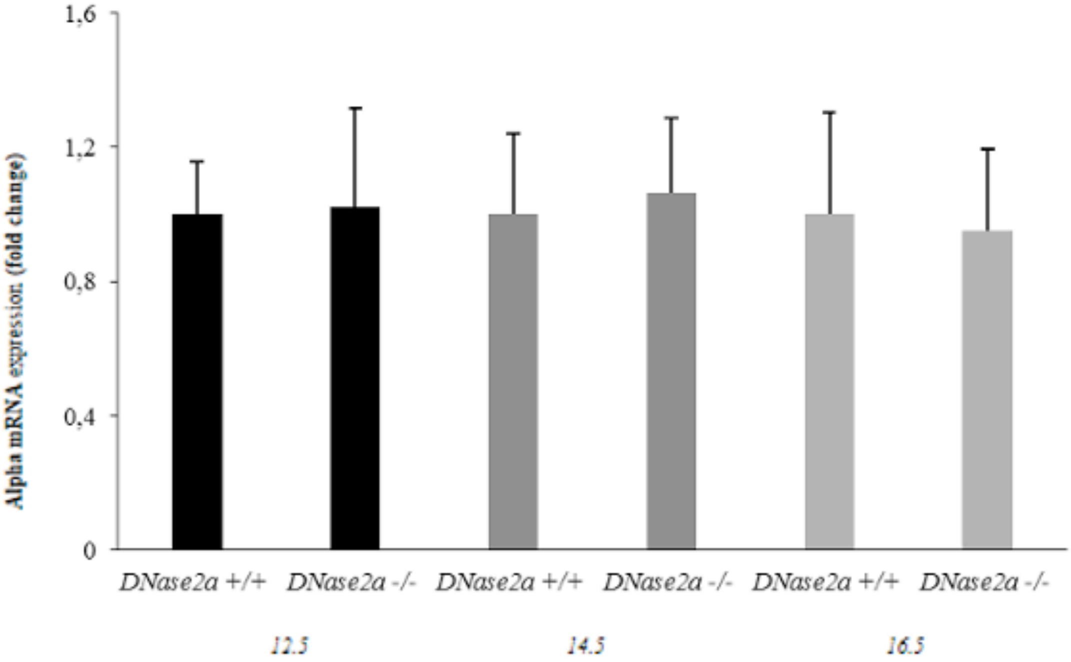

Supplemental Figure 1

Expression level of the mouse alpha globin in *DNase2a* +/+ (n= 4) and *DNase2a* -/- (n = 4) fetal liver at 12.5, 14.5 and 16.5 dpc to investigate whether the *DNase2a* knockout may affect *alpha-globin* gene expression. The results do not show differences in the expression of the alpha globin gene with respect to WT.
mRNA was quantified by RT-qPCR and results are expressed as the value relative to the HPRT mouse levels.

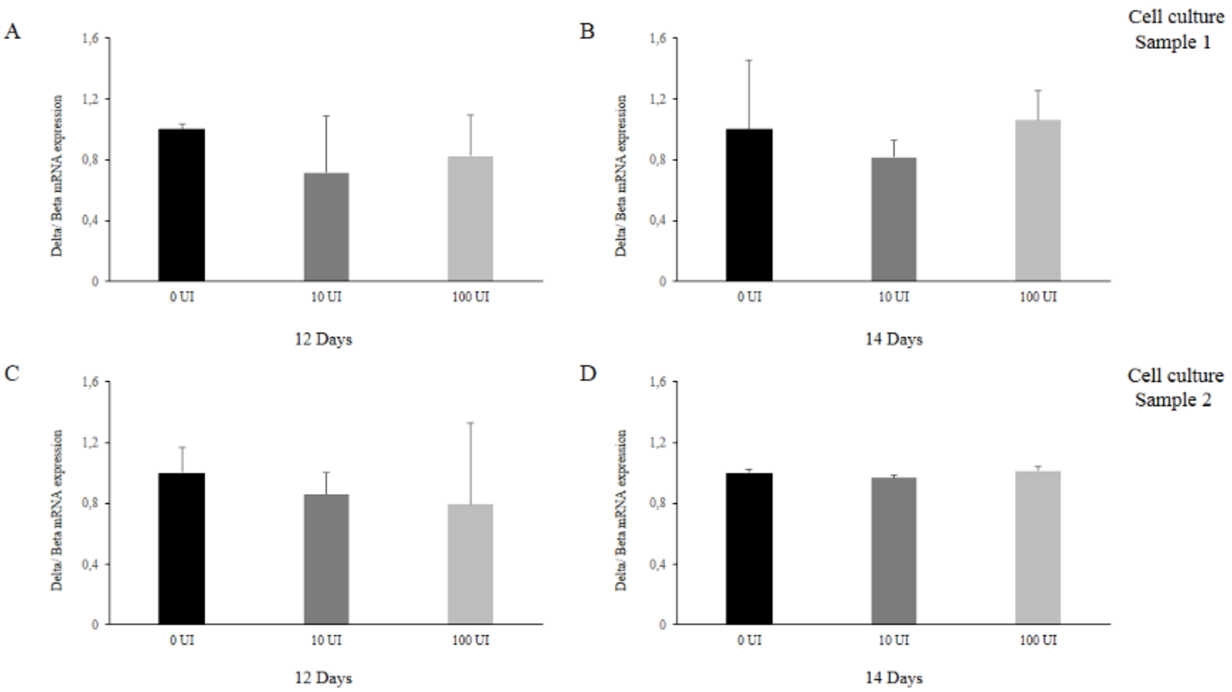

Supplemental Figure 2

Relative mRNA expression level of the human *delta* versus *beta-globin* gene in primary human erythroid cultures at 12 and 14 days after stimulation with 0 UI, 10 UI or 100 UI of IFNb 1a.
mRNA was quantified by RT-qPCR standard techniques.
Panels (A) and (B) show the level of *delta* versus *beta-globin* gene expression in the cell culture sample 1 respectively at 12 and 14 days after stimulation.
Panels (C) and (D) show the level of *delta* versus *beta-globin* gene expression in the cell culture sample 2 respectively at 12 and 14 days after stimulation.
The results do not show statistical differences in the expression of the delta globin gene at 12 and 14 days after stimulation with 0 UI, 10UI or 100UI of IFNb 1a. Stimulation with 1000 UI resulted in cell death after 9 days of culture.

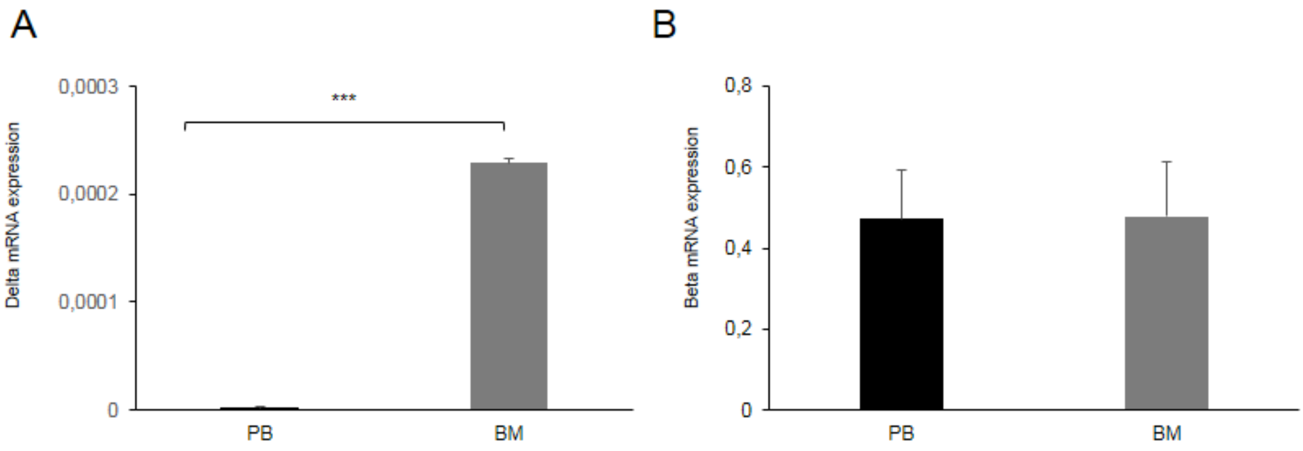

Supplemental Figure 3

Delta and *beta-globin* gene expression level in peripheral blood and bone marrow in ln72 mice (n= 4). mRNA was quantified by RT-qPCR and results are expressed as the value relative to the alpha-globin mouse levels.

(A) Histogram shows the expression level of *delta-globin* gene in peripheral blood and bone marrow.

(B) Histogram shows the expression level of *beta-globin* gene in peripheral blood and bone marrow mRNA. Levels of significance, calculated by Student's t-test, are indicated.

GENE	*SPECIES*	*APPLICATION*	*PRIMER SEQUENCE (5'→3')*
Ifnar1 WT	Mouse	genotyping	AAGATGTGCTGTTCCCTTCCTCTGCTCTGA<br>ATTATTAAAAGAAAAGACGAGGCGAAGTGG
Ifnar1 KO	Mouse	genotyping	CCTGCGTGCAATCCATCTTG<br>ATTATTAAAAGAAAAGACGAGGCGAAGTGG
DNase2a WT	Mouse	genotyping	CAGTGCCACAGAGGACCACT<br>GAGTCTTAGTCCTTTGCTCCG
DNase2A KO	Mouse	genotyping	GATTCGCAGCGCATCGCCTT<br>GAGTCTTAGTCCTTTGCTCCG
Ln72	Human	genotyping	TAAGCCAGTGCCAGAAGAGC<br>TGATACCAACCTGCCCAGG
Gamma globin	Human	RT-qPCR	CTGAGTGAACTGCACTGTGACAAG<br>TCTTTGCCGAAATGGATTGC
Beta globin	Human	RT-qPCR	TTGGACCCAGAGGTTCTTTGA<br>TCACTAAAGGCACCGAGCACT
Delta globin	Human	RT-qPCR	TGAAACCCTGCTTATCTTAAACCAA<br>TTATGTCAGAAGAAAGTGTAAGCAACAG
Alpha globin	Mouse	RT-qPCR	CACCACCCTGCCGATTTC<br>CTCACAGAGGCAAGGAATTTGTC
HPRT	Mouse	RT-qPCR	GCAGTACAGCCCCAAAATGG<br>AACAAAGTCTGGCCTGTATCCAA
HPRT	Human	RT-qPCR	TGACACTGGCAAAACAATGCA<br>GGTCCTTTTCACCAGCAAGCT

Supplemental Table 1

Primers used for genotyping and RT-qPCR.

Type of interferon	Patients after one year treatment (n=47)	Beta carriers (n=9)	Patients before/after treatment (n=25)
IFN beta 1a	55%	45%	52%
IFN beta 1b	45%	55%	24%
IFN beta 1a peg			24%

Supplemental Table 2

Types of interferon administered.

Patients	N. samples	Median hemoglobin levels	5th-95th percentile
Carriers/MS	9	6.4	5.38-6.60
Beta thal carriers (Danjou et al, 2015)	643	5.8	5.0-6.70
MS T0	24	2.6	2.21-3.08
MS T1	24	2.7	2.2 -3.2
IFN beta 1a + IFN beta 1a peg T0	16	2.65	2.28-3.73
IFN beta 1b T0	6	2.80	2.15-3.10
IFN beta 1a + IFN beta 1a peg T1	16	2.85	2.48-4.08
IFN beta 1b T1	6	2.70	2.23-3.20
Beta thal carriers IFN beta 1a	4	6.6	6.43-6.6
Beta thal carriers IFN beta 1b	5	5.8	5.34-6.48

Supplemental Table 3

Characteristics of the studied samples.
Basic descriptive statistic for the patients data, and measurements of HBA2 levels.

Population/ patients	*Hematological parameters*				
MS patients	*RBC*	*MCV*	*MCH*	*RDW*	*HB*
MS T0	*4,74±0,39*	*83,5±9,18*	*27,52±3,39*	*14,8±1,03*	*12,9±0,95*
MS T1	*4,72±0,37*	*81,8±10,21*	*27,1±4,02*	*14,3±1,2*	*12,6±1,25*

Supplemental Table 4

Hematological parameters of the studied samples. None of analyzed parameters showed statistically significant differences between the two groups.
